# A Comparative Analysis of Lead-Free Piezoelectric Micromachined Ultrasonic Transducers for Powered Bio-Sensing

**DOI:** 10.3390/mi17070845

**Published:** 2026-07-16

**Authors:** Alexandru Paolo Mardare, Mamoun Morh, Aldo Ghisi

**Affiliations:** Department of Civil and Environmental Engineering, Politecnico di Milano, Piazza Leonardo da Vinci 32, 20133 Milan, Italymamoun.morh@mail.polimi.it (M.M.)

**Keywords:** pMUT, wireless energy transfer, biosensors, numerical simulation

## Abstract

To exploit ultra-low power logic and architectural design techniques for bio-sensors in the human body, wireless ultrasonic techniques have emerged as a strong candidate for intra-body power transmission, thanks to lower medium attenuation and higher permitted safe intensity levels. When sub-100 μm dimensions are considered for the bio-sensor, most devices struggle to guarantee a suitable voltage and power for digital electronics due to additional scaling requirements. This study investigates three alternative piezoelectric micromachined ultrasonic transducers in aluminum nitride doped with scandium, as reported in the literature, operating in the range 1–10 MHz. Their respective advantages and limitations with regard to energy harvesting and signal transmission performance are analyzed. It is shown that devices with footprints of less than 100 × 100 μm^2^ can achieve voltage outputs of over 150 mV and average power greater than 100 nW.

## 1. Introduction

In recent years, rapid technological progress has been made in the integration of micro-electro-mechanical systems (MEMS) in the biomedical field, driven by the systems’ compact size, low power consumption, and energy-harvesting capabilities [1,2,3]. Thanks to advances in biomedical and microelectronic technologies, it is now possible to implant microdevices inside the human body, as envisaged in, e.g., [4,5]. These devices can be wirelessly powered [6], take local measurements or perform actions relevant to specific therapies or the tracking of biochemical dynamics, and communicate information or action feedback to the exterior.

Among MEMS, piezoelectric micromachined ultrasonic transducers (pMUTs) have emerged as a promising solution for next-generation implantable bio-sensing systems [7,8,9]: by combining MEMS technology with a piezoelectric thin-film layer, micro-scaled devices that operate at MHz frequencies can be designed, making them fast and well suited to coupling with high-impedance fluids. In addition, the piezoelectric properties of the active layer enable the conversion of ultrasound energy into electrical energy and vice versa. For these reasons, pMUTs are widely used in several medical applications, including imaging [10,11], local therapy [8,12,13], and energy harvesting [14]. In the latter field, pMUTs are an excellent alternative to conventional batteries as they supply electrical energy to bio-sensing systems while reducing the occupied area drastically and eliminating the need for periodic surgical replacements [8].

Specifically, this work investigates the energy-harvesting performance of pMUT-based devices for wireless power delivery to implantable medical devices, ensuring compliance with the strict performance and bio-compatibility requirements of the United States Food and Drug Administration (FDA) [15]. Ultrasonic (US) waves are favored over electromagnetic waves due to their superior properties in biological environments, including significantly higher safe exposure limits, lower tissue attenuation (0.5–1 dB/cm·MHz), and shorter wavelengths (approximately 1.5 mm at 1 MHz). These advantages make US-based systems, and pMUT technology in particular, well suited to reliable and efficient power and data transfer in medical applications. The recent use of pMUTs in bio-medicine is also due to the development of biocompatible piezoelectric materials such as aluminum nitride (AlN), which can replace the more widely used and efficient, but lead-contaminated, lead zirconate titanate (PZT), which is typically chosen in microelectronic applications. Notably, the addition of scandium (Sc) to AlN has been found to enhance the effectiveness of piezoelectric energy conversion, providing an acceptable alternative to PZT in biological environments.

Expanding upon the analysis initiated in [16], where only the smallest geometry was investigated, this work focuses on the numerical evaluation of three existing pMUT designs with different size [17], analyzed in both transmitting and receiving configurations, to meet the following performance requirements.

1. Given the toxicity of lead and its incompatibility with biomedical and environmental safety standards, a lead-free alternative to PZT, the most widely used piezoelectric material, must be explored.
2. The device should occupy a footprint of approximately 100 × 100 μm^2^ to ensure suitability for integration into next-generation biomedical implants.
3. The device must be capable of delivering a minimum power of at least 100 nW and generating an output voltage of at least 150 mV, at a distance of 5 mm in a ballistic gel medium.

It is worth mentioning that only objective 1 has been partially studied in the past, while objectives 2 and 3 have not been well explored in the literature.

The paper is therefore organized as follows. Section 2 describes the pMUT geometry and the hypotheses and methods of the three-dimensional numerical simulations, performed with finite elements (FE) via COMSOL Multiphysics^®^, release 6.3. Section 3 summarizes the results of the analyses, carried out in the frequency and transient domain, for different configurations of transmitter (Tx) and receiver (Rx). Section 4 reports the electrical performance of the devices when non-idealities are introduced (e.g., acoustic attenuation). A list of acronyms is provided after the conclusions, collected in Section 5.

## 2. Materials and Methods

### 2.1. Description of pMUT Geometry

**Figure 1:**
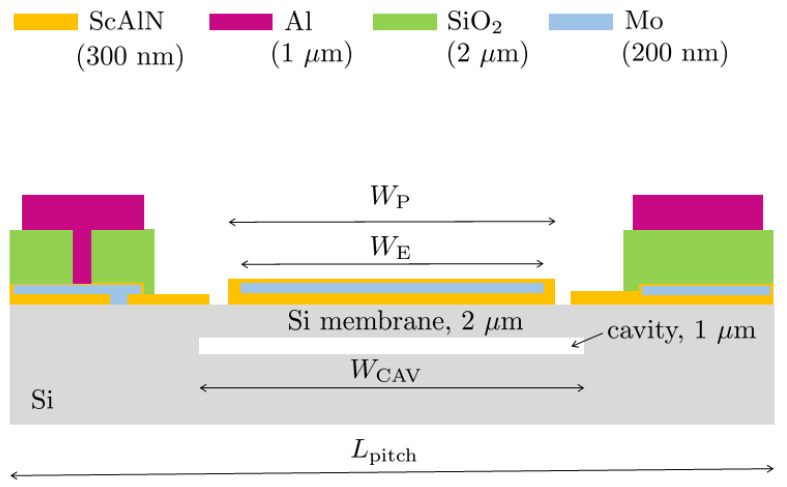
Cross-section area of the device [17], with relevant layer thicknesses.

Among the different pMUT designs presented in the literature, the devices reported in [17] were selected for this study, due to the use of a lead-free and biocompatible material as the piezoelectric layer, as well as the small size and high-frequency operation of each device. The tested pMUT devices, based on the designs reported in [17], feature a square pitch of side $L_{\mathrm{pitch}}$. The piezoelectric layer ($W_{P}$), made of 300 nm thick scandium aluminum nitride (ScAlN), and the top electrode ($W_{E}$), made of 300 nm thick molybdenum (Mo), are square-shaped with rounded corners, having a radius of curvature equal to 70% of their respective widths. The 2 μm thick silicon membrane, which matches the shape of the piezoelectric layer, functions as a structural support layer: phosphorus doping of strategic areas enables it to also act as the bottom electrode. The 1 μm thick cavity, etched on the silicon substrate, follows the geometry of the piezoelectric layer and the top electrode, with width $W_{\mathrm{cav}}$ and similarly rounded corners [17]. From the reference article, three of the six presented devices were selected for our numerical simulations because of their operating frequencies, which are optimal for water-coupled applications and hence supposedly acceptable for intra-body operation. The geometric parameters of the selected devices are summarized in Table 1 and illustrated in Figure 1.

### 2.2. Material Properties

In the structural domain describing the pMUT, Mo is isotropic elastic, Si and ScAlN are anisotropic elastic, ScAlN is also linearly piezoelectric. The elastic constants for silicon and the Young’s modulus for Mo are assumed according to [18].

It is worth mentioning that, among piezoelectric materials, PZT consistently shows the highest performance especially for transmitting devices, thanks to its larger piezoelectric coefficients that provide a higher electroacoustic conversion efficiency. However, Sc_0.15_Al_0.85_N emerges as a promising alternative, offering a good trade-off between piezoelectric performance, CMOS compatibility, and biocompatibility, the latter property not belonging to PZT due to its lead content. Table 2 compares a relevant piezoelectric coefficient between the PZT and the AlN, along with the doped variant ScAlN considered in this work.

The key piezoelectric properties of Sc_0.15_Al_0.85_N are derived from [20], where density functional theory simulations and experimental validation are presented and the properties are provided as a function of the Sc percentage. The dielectric constant $\varepsilon_{33}$ is set to 13.79, while the mass density was estimated as ρ = 3230 kg/m^3^. For the piezoelectric coefficients, in the *e*-form, they can be expressed as follows: $e_{31}$ = −0.63 C/m^2^, $e_{33}$ = 1.77 C/m^2^, $e_{15}$ = −0.29 C/m^2^. The anisotropic elastic compliances for Sc_0.15_Al_0.85_N are collected in Table 3.

For the ballistic gel medium, a density of 1060 kg/m^3^ and a sound velocity of 1560 m/s were assumed. The assumption of homogeneous gel behavior is made in anticipation of future experimental tests. However, if the tissue exhibits significant heterogeneity, the properties of the acoustic medium should be modeled accordingly.

### 2.3. Methodology

The performance of the pMUTs in ballistic gel was characterized through numerical simulations, involving several steps. Firstly, the pMUT was modeled using FE analysis to evaluate its operating frequency and its frequency response spectrum in ballistic gel. Each Rx was evaluated as a single element and also as a 2 × 2 array for the R19, with a focus on maximizing the voltage generated and the energy stored in the piezoelectric layer. Secondly, various Tx layouts were tested to determine the minimum configuration required, specifically, the array size, to generate the desired amount of acoustic power. Thirdly, based on previous results, a time-domain analysis was performed using the Rx as a single device (or configured in a 2 × 2 array for R19) and the Tx as an 8 × 8 array.

A sinusoidal voltage with an amplitude of 20 V was applied to each pMUT element of the Tx array, possibly with a duty cycle selected to comply with one of the FDA limits on US waves, namely, a power density of $720\;\mathrm{mW}/{\mathrm{cm}}^{2}$. However, other FDA regulations, such as those relating to the mechanical index, which quantifies the risk of cavitation in body fluids (defined as the ratio of the peak negative pressure to the square root of the ultrasound frequency), are not considered here.

**Figure 2:**
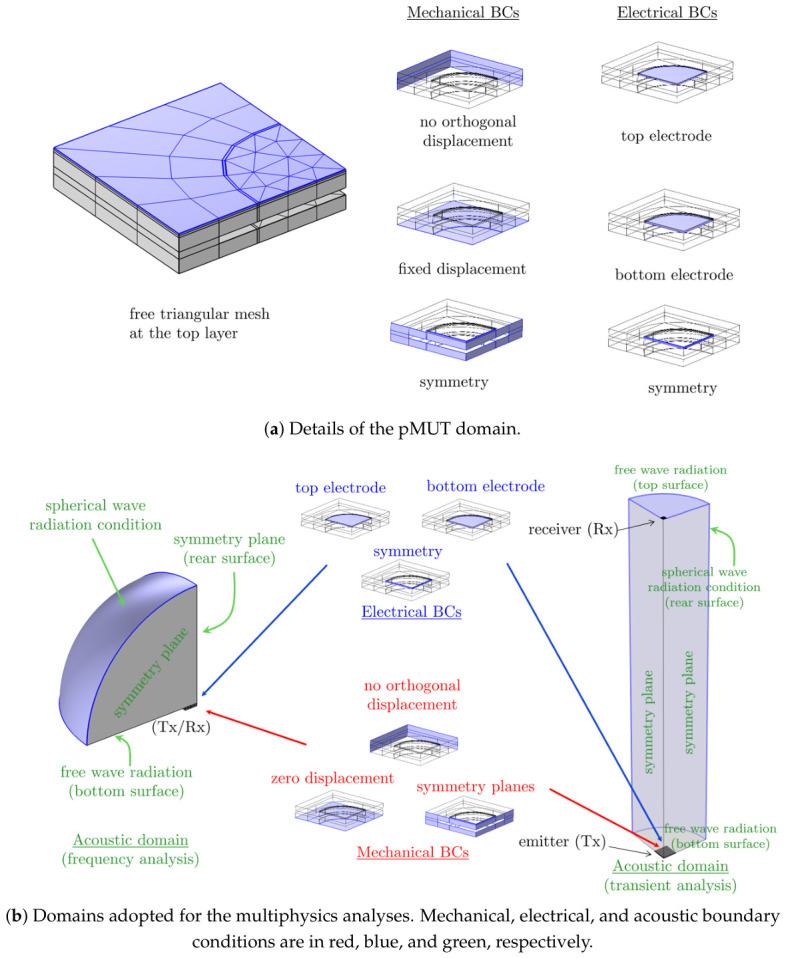
Schematic representation of adopted boundary conditions (**a**) for the pMUT only and (**b**) for the acoustic domains during frequency and transient analysis.

The pMUT meshing strategy uses a combination of three-dimensional solid and shell elements to strike a balance between accuracy and computational efficiency [21]. Due to the high aspect ratio of the thin films, shell elements are used for most of the structure, while the active piezoelectric layer is modeled with 3D solid elements to capture its full electro-mechanical response. First, the piezoelectric layer is discretized on its surface using a free triangular mesh (see Figure 2a). This mesh is then refined to one-third of the top electrode radius and swept to form prismatic elements. The external plates use third-order Lagrangian shell elements and the acoustic domain is meshed with second-order tetrahedral elements. The lateral size of these elements is set to one-fifth of the acoustic wavelength to ensure there are around 10 nodes per wavelength for accurate wave propagation modeling [21].

The mechanical boundary conditions, as shown in Figure 2a (center), include symmetry planes, a fixed (zero displacement) support base, and impose zero orthogonal displacement at the lateral surfaces of the single or pMUT array. The top and bottom electrode surfaces are indicated in Figure 2a (right) for the (piezo)electric field. In the acoustic domain adopted for the frequency analysis, see Figure 2b (left), the curved surface of a quarter of a sphere presents a spherical wave radiation condition to prevent spurious reflections; the base surface allows free wave radiation; symmetry boundary conditions are applied to the vertical flat surfaces of the quarter of a sphere. For the acoustic transient analysis, see Figure 2b (right), the considered geometry is a quarter-cylinder domain, which still captures the relevant interactions between the Tx and the Rx. To prevent spurious reflections, a spherical radiation boundary condition is applied to the curved surface of the quarter-cylinder; the top and bottom surfaces present free radiation conditions; symmetry conditions are applied to the flat vertical surfaces.

A typical transient analysis contains about 200,000 elements and half a million degrees of freedom and occupies for 10 h a workstation Intel Xeon W-2275 CPU @ 3.30 GHz and 128 GB RAM.

## 3. Results

### 3.1. Receivers

**Figure 3:**
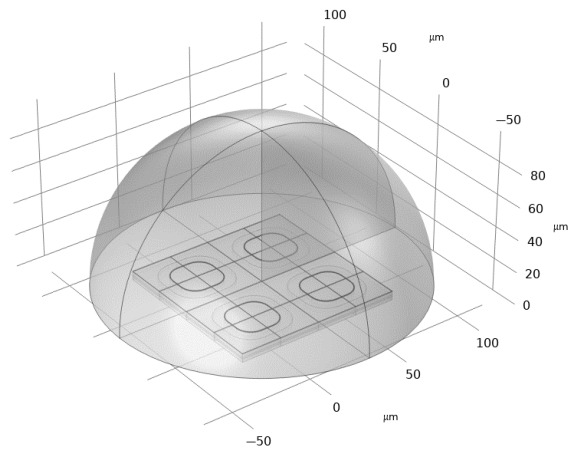
R19 2 × 2 array configuration within ballistic gel.

This section presents the performance of the pMUTs in receiving mode, with the setup depicted in Figure 2b (left) and in Figure 3 for the 2 × 2 R19 array case. Ballistic gel was selected as the acoustic medium for this and subsequent analyses due to its acoustic properties (density, speed of sound, and attenuation) closely mimicking those of human tissue [22].

To this end, a frequency domain analysis was performed, involving the application of a reference acoustic pressure of 1 kPa directly to the pMUT surface, and the evaluation of the resulting voltage at the top electrode, as well as the energy stored in the piezoelectric layer.

**Figure 4:**
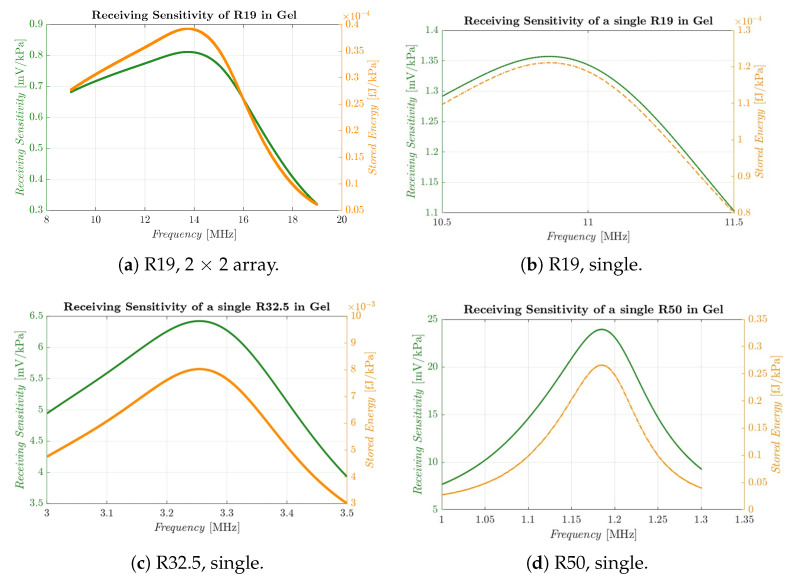
Receiving sensitivity in ballistic gel for the considered pMUT configurations.

As one R19 pMUT occupies a minimal area of 50 × 50 μm^2^, enabling the implementation of a 2 × 2 array without exceeding the specified constraints, the 2 × 2 R19 array configuration was also considered, in addition to the single R19, R32.5, and R50 cases. Although a small amount of voltage is lost with respect to the single pMUT due to cross-talk phenomena, the array configuration theoretically enables up to four times more energy to be harvested and the overall impedance to be reduced by one quarter, while maintaining the same sensed voltage across all elements, by connecting the four pMUTs in parallel. Frequency domain analysis revealed a receiving sensitivity of 0.8 mV/kPa at a resonance frequency of 13.7 MHz, as shown in Figure 4a.

This result is slightly lower than that for the single R19, shown in Figure 4b, where a sensitivity of approximately 1.35 mV/kPa was obtained; it is also worth noting the shift in resonance frequency, which corresponds to 10.5 MHz, approximately 3 MHz lower than the 2 × 2 array case. This demonstrates the significant influence of acoustic and mechanical cross-talk on the overall behavior of the system.

The plots in Figure 4c,d show the receiving sensitivity for the R32.5 and R50, respectively. In both cases, it is significantly higher than for the R19, ranging from approximately 6.5 mV/kPa for the R32.5 to approximately 24.0 mV/kPa for the largest R50. This outcome is expected as the thickness of the layers remains fixed while the in-plane dimensions increase; the largest pMUTs are most deformable under a given acoustic pressure wave and therefore generate the greatest potential difference. However, the operating frequency changes considerably, from around 3.25 MHz for the R32.5 to 1.16 MHz for the R50. The receiving bandwidth also varies significantly, being larger for the R19 at around 2 MHz and diminishing as the pMUT dimensions increase to 0.8 MHz for the R32.5 and 0.2 MHz for the R50.

Finally, Figure 4 reports the stored energy as a function of frequency and imposed pressure, confirming that the largest pMUT acquires the most energy for a given wave cycle. However, this information must be considered alongside the operating frequency of individual pMUTs, as will be seen in the next Section 4.

### 3.2. Transmitters

Following preliminary analyses that are not reported here, the choice of an 8 × 8 array for the Tx is driven by the need to achieve sufficient input acoustic pressure, to provide adequate power to the Rxs, whose sensitivities are shown in the previous Section 3.1. Furthermore, the objective is to highlight the distinct roles of each pMUT within the considered set as either Tx or Rx. However, increasing the array size introduces significant challenges in compact devices, such as managing cross-talk between adjacent pMUTs. The behavior of the Tx was studied through a frequency domain analysis, by measuring the emitted pressure at a distance equal to five wavelengths (corresponding to approximately 0.5 mm, 2.4 mm, and 6.5 mm for R19, R32.5, and R50, respectively), when an input voltage of 1 V is applied to the top electrode. This distance was chosen to ensure that the emitted pressure is measured in the far-field region, where the acoustic wave is stable and unaffected by interference.

**Figure 5:**
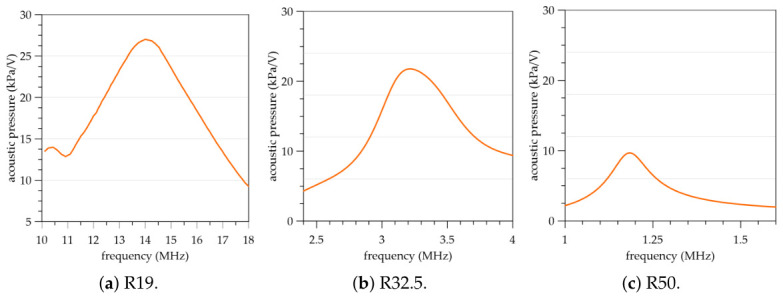
Acoustic pressure generated by the 8 × 8 array measured at a distance equal to five wavelengths in a gel medium.

Indeed, at the operating frequency of the 2 × 2 R19 array as Rx, the array can emit an acoustic wave with a sensitivity of approximately 26 kPa/V at an operating frequency of approximately 14.0 MHz. As illustrated in Figure 5a, the large bandwidth of the 8 × 8 R19 array enables optimal acoustic pressure to be generated even at slightly different frequencies, e.g., lower than the resonance peak. At the operating frequency of the single R19, 10.5 MHz, the acoustic pressure is lower at around 14 kPa/V.

As the dimension of the pMUT increases, from R32.5 (Figure 5b) to R50 (Figure 5c), the operating frequency of the Tx remains conveniently aligned with the corresponding frequency of the Rx. However, the acoustic pressure generated by unit voltage for the 8 × 8 array Tx decreases, first slightly in the R32.5 case, down to approximately 22 kPa/V, then significantly in the R50 case, down to less than half that of the R19 case at approximately 10 kPa/V.

### 3.3. Transient Analysis of the Tx-Rx System

**Figure 6:**
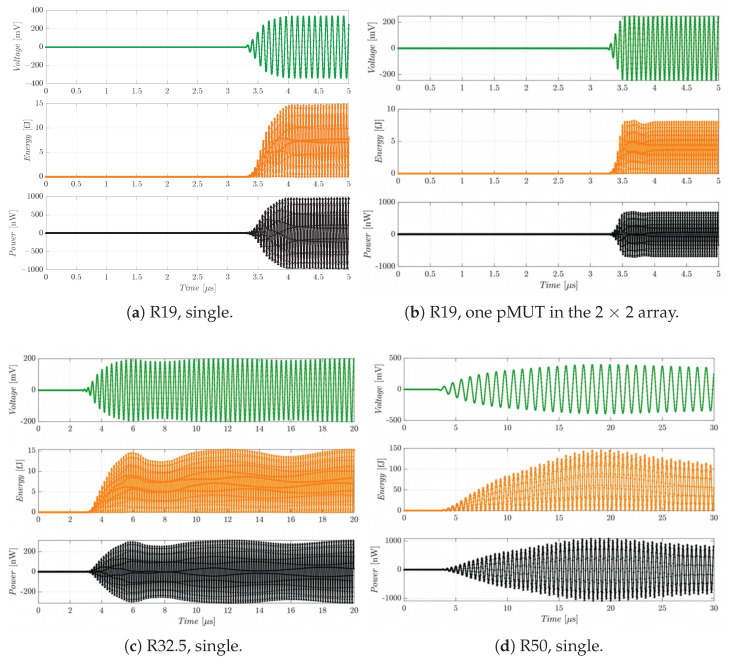
Voltage, energy, and instantaneous power by the various pMUTs.

The core of this analysis is to quantify the amount of power harvested in the ballistic gel and to verify the transient response using the Txs and Rxs presented in the previous two sub-sections. For reference, the full 8 × 8 array is driven with a sinusoidal input voltage, with an amplitude of 20 V at frequencies of 13.7 MHz, 3.25 MHz, and 1.16 MHz for R19, R32.5, and R50, respectively. All elements operate in phase. As shown in Figure 2b, symmetry is exploited to reduce the computational burden without compromising the accuracy of the results. After the acoustic wave has traveled for a certain time, the Rx starts to vibrate and, thanks to the piezoelectric effect, begins to accumulate charge as soon as the acoustic wave generated by the Tx reaches it. Given that the speed of sound in ballistic gel is 1560 m/s, the pressure wave emitted by the array takes approximately 3.21 μs to travel 5 mm to the Rx, which is consistent with what is observed, e.g., in Figure 6a.

The time evolution of the electrical performances obtained during the time-domain analysis for each Rx is represented in Figure 6; the maximum amplitudes are collected in Table 4. For the 2 × 2 array R19, the power obtained from one pMUT was multiplied by four, assuming an electrical connection in parallel. The settling times for R19 pMUTs appear to be of the order of one μs, while they are longer for R32.5 and R50. For the latter two, the measurements indicated in the table should be considered with a larger margin of error, even though the trend is clear, since the steady-state condition is not perfectly reached in the simulations, which were kept at reasonable wall-clock time and limited to a duration of 20 μs and 30 μs for R32.5 and R50, respectively. Finally, it is worthwhile emphasizing that these results have to be considered ideal, since they do not account for the FDA requirements or the attenuation of the pressure waves in the medium. These further effects are discussed in the next section, Section 4.

## 4. Discussion

This section discusses critical physical effects and engineering requirements that are not directly captured by the FE modeling. Specifically, the discussion covers safety limits for acoustic radiation in the human body, signal attenuation within the ballistic gel medium, and internal mechanical stress in the pMUT. Additionally, the average power is evaluated by assuming a reference resistive load connected to the pMUT. Finally, a comparative analysis of the different pMUT geometries is presented at the end of the section.

Due to the high transmission sensitivity of the pMUTs, specifically, the R19 variants, the spatial peak temporal average intensity $I_{\mathrm{SPTA}}$ must be evaluated against FDA safety limits. It is expressed as follows [15]:

(1) $$I_{\mathrm{SPTA}}=\frac{p^{2}}{2\rho c}\cdot \eta ,$$

where *p* is the peak sound pressure, ρ and *c* are the density and speed of sound of the ballistic gel, respectively, and η is the signal duty cycle.

For the 2×2 R19 array under continuous-wave excitation and a 20 V driving voltage, the maximum intensity occurs at a distance of 0.5 mm due to Rayleigh distance considerations, yielding an $I_{\mathrm{SPTA}}$ of $8300\;\mathrm{mW}/{\mathrm{cm}}^{2}$. Consequently, a maximum duty cycle of 8.5% is required to comply with FDA safety limits. In principle, the high operating frequency of the Rx would enable the system to rapidly reach a steady state and harvest sufficient energy within a short time frame, but the strong reduction in the operation because the duty cycle limits the usefulness of the 2×2 R19 array. The single R19 Rx exhibits an $I_{\mathrm{SPTA}}$ of $2350\;\mathrm{mW}/{\mathrm{cm}}^{2}$, requiring a maximum duty cycle of 30%, better than the array but still demanding.

The R32.5 and R50 Txs, instead, produce $I_{\mathrm{SPTA}}$ values of 206 and $0.67\;\mathrm{mW}/{\mathrm{cm}}^{2}$, respectively. Because both values fall well below the FDA limit of $720\;\mathrm{mW}/{\mathrm{cm}}^{2}$, no duty cycle constraints are required, a significant advantage.

US waves propagating through the ballistic gel medium experience acoustic dissipation [23], which dampens the pressure wave amplitude. This attenuation directly degrades Rx performance and must be quantified to accurately evaluate pMUT behavior. In this study, the attenuation coefficient of the ballistic gel is applied to the acoustic pressure measured at the Tx, see Figure 5, by assuming conservatively a coefficient of reduction of 1.07dB/(MHz·cm) (consistent with [24] and representative of thick human tissue [22]). Consequently, total attenuation depends heavily on both the device operating frequency and the transmission distance. More precisely, the attenuation *a* in decibels can be expressed as $a=1.07\cdot f_{0}\cdot d$, where $f_{0}$ is the operating frequency in MHz and *d* is the distance in cm. Considering the respective operating frequencies of the Rxs, the attenuation at a reference distance of 5 mm is 47.6% for the single R19 and 57% for the 2×2 R19 array. For the R32.5 and R50 variants, which operate at much lower frequencies, the attenuation drops significantly to 18% and 7%, respectively. Table 5 summarizes the adjusted electrical performance parameters after accounting for acoustic attenuation.

Another critical parameter is the von Mises stress induced by the acoustic pressure reaching the Rx. If the peak stress exceeds the material yield strength, the top electrode will undergo plastic deformation, potentially leading to mechanical failure. Transient numerical simulations indicate a maximum von Mises stress of $\sigma_{\mathrm{VM},\max}\approx 39.9\;\mathrm{MPa}$ at the center of each R19 Rx pMUT. This value is well below the yield strength of the material ($\sigma_{\mathrm{yield},\mathrm{Mo}}\approx 0.55\;\mathrm{GPa}$), ensuring that the pMUT operates strictly within its elastic regime, even under elevated driving voltages. The remaining layers of the pMUT exhibit even lower stress levels. For larger pMUT designs, stress levels are negligible as they are substantially lower than those observed in the R19 configuration.

Table 5 compares the performance of the different pMUT Rxs. As established in [16], the stored energy per cycle scales as $E\propto CV^{2}$ (where *C* is capacitance and *V* is voltage); consequently, the energy stored by the R50 variant is approximately two orders of magnitude greater than that of the R19. The R32.5 configuration harvests several times more energy than the R19 variants, though it remains within the same order of magnitude.

Because the time-domain plots in Figure 6 correspond to a piezoelectric device connected to a purely capacitive circuit, the average power must be evaluated across a reference resistive load to characterize the energy-harvesting capacity of each device. For a unit resistance, the steady-state average power can be defined as $P_{\mathrm{average}}=V_{0}^{2}/2$, where $V_{0}$ is the steady-state sinusoidal voltage amplitude after accounting for attenuation (see Table 5). Incorporating the duty cycle yields the effective average power, $P_{\mathrm{average},\;\mathrm{effective}}=\eta \cdot P_{\mathrm{average}}$. The resulting average power values for a 100kΩ load are listed in Table 5 for the various Rxs. Notably, the duty cycle constraint imposes a severe limitation on the smaller R19 variants. Required for both the single and array configurations, this constraint significantly reduces the time-averaged availability of the voltage and power outputs.

The average power levels are adequate for digital electronics only in the case of the R32.5 and R50 variants; the output voltage and power for both the single R19 and the R19 array fall short of the design objectives. The R32.5 design yields a post-attenuation voltage output of 163 mV, which is comparable to that of the single R19. However, it provides a substantially higher average power and requires no duty cycle restrictions. This is a critical advantage for the design of the associated power-management and digital electronics, easily compensating for its slightly lower voltage compared to the single R19. The R50 achieves a remarkable voltage output of 324 mV. Consequently, while downsizing to a high-frequency pMUT, such as happens with the R19, increases power density and Rx sensitivity, it introduces strict duty cycle limitations to comply with FDA regulations. Furthermore, because acoustic attenuation scales with frequency, the operational range of high-frequency pMUTs is inherently restricted.

These performance metrics must be balanced against the physical footprint of each device, as listed in the final row of Table 5. Given that the long-term objective is device miniaturization for intra-body biosensors, physical size heavily influences the selection of the optimal pMUT configuration. Accordingly, while the R50 delivers superior energy-harvesting performance and is the least affected by acoustic attenuation, its dimensions slightly exceed the area constraints specified in Section 1.

Summarizing the results, the single R19 design in principle enables rapid operating speeds and provides a high power density relative to its size due to its high resonance frequency, but in practice, attenuation in the ballistic gel significantly degrades its performance. To maintain acceptable voltage and power levels, the duty cycle must be restricted to less than 10% for the array and approximately 30% for the single element. This constraint complicates the power management circuitry and curtails the operational distance between the Tx and Rx. The R32.5 strikes an optimal trade-off between these designs, despite its voltage falling below 200 mV once acoustic attenuation is factored in.

Finally, to contextualize the magnitude of the technical challenges involved, it is instructive to compare the power transmission efficiency (PTE) of these pMUTs with state-of-the-art devices reported in the literature. PTE is defined as the ratio between $P_{\mathrm{in}}/P_{\mathrm{out}}$, where $P_{\mathrm{in}}=I_{\mathrm{SPTA}}\cdot A$ (*A* being the Tx footprint), and $P_{\mathrm{out}}$ is the average power, possibly accounting for the duty cycle. In the table, the input and output powers are given per unit area. As summarized in Table 6, scaling down the device dimensions inherently elevates the operating frequency. This, in turn, increases acoustic medium attenuation and brings the system closer to the FDA exposure limits for emitted radiation. Although the performance values per unit area are (sometimes significantly) lower than those reported in the literature, it is important to emphasize that the primary challenge for future intra-body applications is to guarantee the baseline voltage and average power thresholds required to reliably power downstream digital electronics rather than to provide PTE comparable to that in other application fields.

## 5. Conclusions

This work presented a numerical study of several pMUT configurations reported in the literature for wireless energy harvesting in biomedical applications. These configurations were evaluated against strict design requirements, including FDA acoustic radiation limits, a footprint below $100\times 100\;\mu m^{2}$, and biocompatibility. ${\mathrm{Sc}}_{0.15}{\mathrm{Al}}_{0.85}N$ proved to be an excellent lead-free, CMOS-compatible alternative to PZT in terms of piezoelectric performance. Furthermore, given the inverse relationship between transmission and receiving sensitivity, a smaller ScAlN pMUT exhibits superior receiving capabilities, making it highly suitable for energy-harvesting applications.

Three alternative designs, designated as R50, R32.5, and R19, were compared at operating frequencies of 1.16 MHz, 3.25 MHz, and above 10 MHz, respectively. Although miniaturization enables high-frequency operation (>10MHz for R19), it also introduces increased cross-talk (a stiffening effect) between array elements, requires strict duty cycle constraints (8.5% for the 2×2 R19 array and 30% for the single R19 element), and exacerbates acoustic wave attenuation over distance. Despite having an area 25% larger than the specified design requirements, the largest device, R50, performs well due to its lower operating frequency and greater robustness, though it requires longer excitation bursts to reach a steady state. The intermediate-sized device, R32.5, avoids severe attenuation of the acoustic signal while achieving acceptable energy-harvesting performance, while maintaining a footprint of $75\times 75\;\mu m^{2}$ that remains within the design limit.

Consequently, the resonance frequency of the device proves to be a critical parameter for energy-harvesting applications. Although high frequencies offer the advantage of enabling high-speed operation, they also lead to significant wave attenuation in the ballistic gel because attenuation is frequency-dependent. The latter aspect can induce substantial energy losses; mitigating this effect will be a primary focus of future developments. Indeed, it is worth underscoring the dramatic increase in operating frequency between the R32.5 and R19 designs, which leaps from 3.25 MHz to approximately 14 MHz—a jump of more than 10 MHz. Therefore, it is highly practical to tune the operating frequency to precisely match the required electrical performance for such micro-scale applications. This will require the redesign and fabrication of a new pMUT geometry aiming to achieve a working frequency below 5 MHz with the smallest possible footprint allowed by micro-fabrication limits. Starting from the R32.5 dimensions, further size reductions can only be achieved by decreasing the thickness of the membrane layers to effectively manage the operating frequency. A non-incremental development would consider completely different geometries, even curved ones such as in [26].

Future work will focus on the fabrication and experimental validation of optimized pMUTs using silicon-on-nothing processes. Additionally, simulations will be expanded to incorporate nonlinear, thermal, and viscoelastic effects. Experimental evaluations will also be conducted to assess residual stresses, wave attenuation in ballistic gel and actual biological media, and fatigue strength of the Rx devices, alongside the monolithic or heterogeneous integration of the MEMS pMUT array with CMOS circuitry.

## Figures and Tables

**Table 1:** Geometrical parameters of the tested devices.

Device	$W_{\mathrm{CAV}}$	$W_{E}$	$L_{\mathrm{pitch}}$	$W_{\mathrm{piezo}}$
Name	(μm)	(μm)	(μm)	(μm)
R50	100	70	125	70
R32.5	65	45	75	45
R19	38	26	50	26.6

**Table 2:** Comparison of $d_{33}$ coefficient and CMOS compatibility for different piezoelectric materials.

Material	$d_{33}$ (pC/N)	CMOS Compatible
PZT	60–130	Limited (but improving: [19])
AlN	5.5	Yes
Sc_0.15_Al_0.85_N	15–20	Yes

**Table 3:** Elastic compliances of Sc_0.15_Al_0.85_N, with $C_{66}=\frac{1}{2}\left(C_{11}-C_{12}\right)$.

Compliance Coefficient	Value (GPa)
$C_{11}$	348.97
$C_{12}$	144.97
$C_{13}$	119.68
$C_{33}$	305.95
$C_{44}$	104.82
$C_{66}$	102.00

**Table 4:** Extracted electrical performance from the transient simulation in ideal conditions (maximum values).

Quantity (Unit)	Single R19	2 × 2 Array R19	R32.5	R50
Voltage (mv)	337	213	199	348
Energy (fJ)	15	32.2	14.5	115
Instantaneous power (nW)	960	2740	250	840

**Table 5:** Extracted electrical performance from the transient simulation when attenuation effects were accounted for.

Quantity (Unit)	Single R19	2 × 2 Array R19	R32.5	R50
Voltage (mv)	177	92	163	324
Energy (fJ)	4	6	14.5	115
Duty cycle	30%	8.6%	no	no
Average power (nW)	47	4	132	525
Area (μm^2^)	50 × 50	100 × 100	75 × 75	125 × 125

**Table 6:** Comparison of power transfer efficiency (PTE), after accounting for attenuation and duty cycle. The material used in this paper, Sc_0.15_Al_0.85_N, is indicated by *.

Parameter	Ref. [25]	Ref. [14]	R19	R32.5	R50
Piezoelectric material	AlN	Sc_0.096_Al_0.904_N	*	*	*
Acoustic medium	water	water	gel	gel	gel
Operating frequency (MHz)	3	0.98	13.7	3.25	1.16
Transfer distance (mm)	25	20	5	5	5
Input power density (mW/mm^2^)	7	7.05	88	70	12
Output power density (μW/mm^2^)	16.5	164	20	24	34
Receiver footprint area (mm^2^)	2.55	3.2	0.0025	0.0056	0.0156
Transducer thickness (μm)	0.45	1.0	0.30	0.30	0.30
PTE (%)	0.236	2.33	0.021	0.034	0.28

## Data Availability

Data can be obtained by kindly writing to the corresponding author.

## Abbreviations

The following abbreviations are used in this manuscript:

AlN	aluminum nitride
CMOS	complementary metal-oxide semiconductor
FE	finite element
FDA	Food and Drug Administration
Mo	molybdenum
pMUT	piezoelectric micromachined ultrasonic transducer
PTE	power transfer efficiency
PZT	lead zirconate titanate
Rx	receiver
ScAlN	scandium aluminum nitride
Si	monocrystalline silicon
SiO_2_	silicon dioxide
Tx	transmitter
US	ultrasonic
